# Four-fold increase in solar forcing on snow in western U.S. burned forests since 1999

**DOI:** 10.1038/s41467-019-09935-y

**Published:** 2019-05-02

**Authors:** Kelly E. Gleason, Joseph R. McConnell, Monica M. Arienzo, Nathan Chellman, Wendy M. Calvin

**Affiliations:** 1 Division of Hydrologic Sciences, Desert Research Institute, 2215 Raggio Parkway, Reno, NV 89512 USA; 2 Department of Environmental Science and Management, Portland State University, Portland, OR 97207-0751 USA; 3 Geological Sciences and Engineering, University of Nevada, Reno, 1664 N. Virginia Street, Reno, NV 89557 USA

**Keywords:** Fire ecology, Hydrology

## Abstract

Forest fires are increasing across the American West due to climate warming and fire suppression. Accelerated snow melt occurs in burned forests due to increased light transmission through the canopy and decreased snow albedo from deposition of light-absorbing impurities. Using satellite observations, we document up to an annual 9% growth in western forests burned since 1984, and 5 day earlier snow disappearance persisting for >10 years following fire. Here, we show that black carbon and burned woody debris darkens the snowpack and lowers snow albedo for 15 winters following fire, using measurements of snow collected from seven forested sites that burned between 2002 and 2016. We estimate a 372 to 443% increase in solar energy absorbed by snowpacks occurred beneath charred forests over the past two decades, with enhanced post-fire radiative forcing in 2018 causing earlier melt and snow disappearance in > 11% of forests in the western seasonal snow zone.

## Abstract (web summary)

The impacts of forest fire activity in the western US on snow melt are poorly quantified. Here the authors use satellite and field-based observations to document a four-fold increase in the solar forcing on snow in western burned forests from 1999 to 2018.

## Introduction

Most annual precipitation falls as snow in the American West^1^, with mountain snowpacks serving as water reservoirs that recharge aquifers and sustain streamflow into drier summer months^2–4^. Snow is a particularly important water resource in the Intermountain West where 50–70% of precipitation is seasonally stored as snowpack. Rising air temperatures have reduced recent snowpack volume and associated seasonal snow-water storage, resulting in accelerated snowmelt and earlier springtime meltwater release^5–8^, which ultimately threatens the timing and volume of downstream water resource availability^8,9^. Earlier snowmelt extends the growing season resulting in amplified late summer drought^10^, reduces forest productivity limiting carbon sequestration^11^, and shifts phenological synchronicity with impacts to the reproductive success of many plants, pollinators, birds, and fish^12,13^. Another consequence of climate warming and earlier snowmelt has been an increase in forest fire intensity, duration, extent, and frequency^14–16^, with total area burned likely to continue increasing across the West^17–20^. The headwater regions of the Rocky Mountains are especially vulnerable, with an anticipated 300–700% increase in burned area for every 1 °C increase in global average temperature^21^.

The vast majority of western forest fires occur in the seasonal snow zone, and such fires result in spatial and temporal changes in snow accumulation, ablation, and melt^22–26^. Removal of the canopy by fire results in reduced interception and enhanced snow accumulation^23,25^, but the more open canopy also results in more incident sunlight on the snowpack surface^22,24^, as well as changes in longwave radiation and turbulent energy fluxes^22^. In addition, increased deposition of light absorbing impurities (LAI) from the charred canopy results in reduced snow albedo^24,25^. As a result of these changes collectively referred to here as post-fire radiative forcing on snow, forest fires lead to mid-winter loss of snowpack volume^27^, accelerated snowmelt, and earlier snowpack disappearance^25,28,29^.

The magnitude and persistence of LAI-related albedo changes, as well as their associated radiative impacts on snow-water resources over broad scales, are presented in the current study. The extent of burned forests in the seasonal snow zone has dramatically increased across the West. While in these burned forests, 5 day earlier snow disappearance persists for >10 years following fire. Fire-related impurities, specifically black carbon and burned woody debris, darkens snowpack and lowers snow albedo for 15 winters following fire. A four-fold increase in the solar forcing on snow in western burned forests occurred from 1999 to 2018. Future increases in forest fires under a warming climate and associated radiative forcing potentially will have vast implications for the volume and timing of western streamflow and therefore water resource management^30,31^.

## Results

### Snow disappears earlier after forest fire across the West

**Fig. 1:**
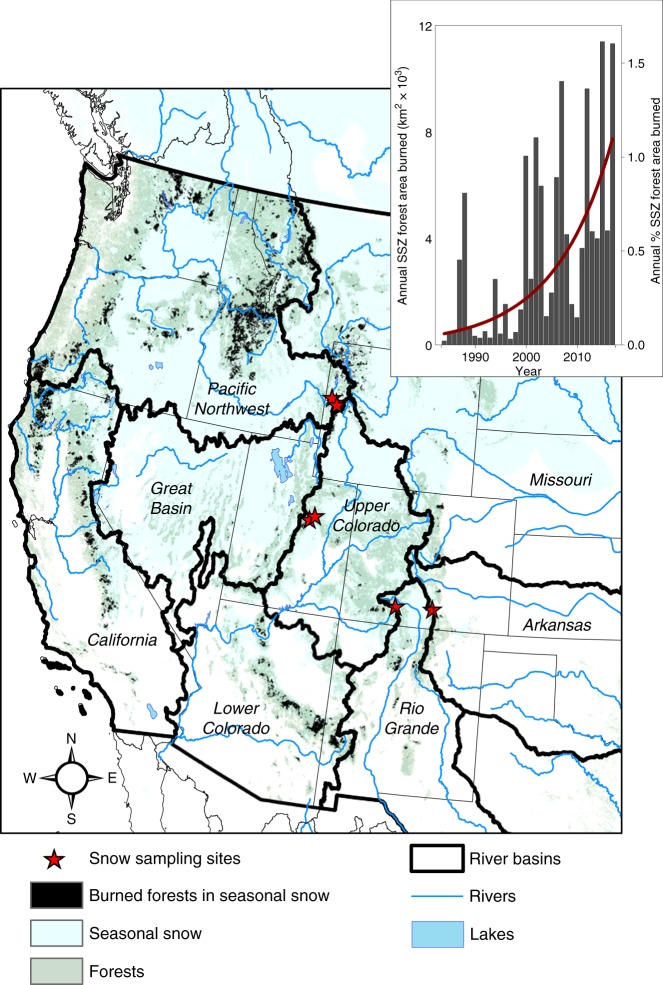
Locations of forest fires in the western seasonal snow zone (SSZ) and total area burned (inset) from 1984 to 2017. Area burned determined from Landsat imagery has increased at an average rate of up to 9% per year in recent decades as a result of climate warming and a legacy of fire suppression. Also shown are 2017 snow sample collection sites located in seven recently burned forests (burned 1–15 years prior to snow sampling)

**Fig. 2:**
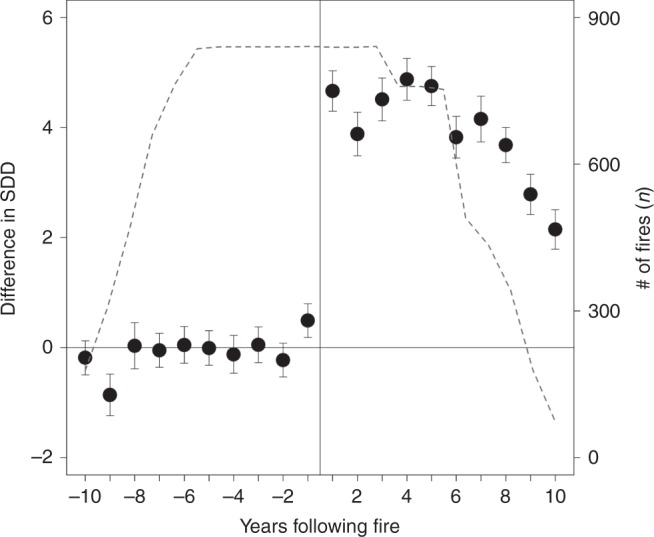
Change in mean snow disappearance date (SDD) before and after fire. SDD was evaluated for all burned forests (*n* = 841; dashed line) located in the western U.S. seasonal snow zone using 2000–2016 MODIS satellite measurements. Forest fire resulted in a clear and immediate shift in SSD, with impacts of fire starting to decline after ~8 years but persisting for >10 years. Error bars indicate the standard error of the mean

Satellite-based observations from 1984 to 2017 document a marked change in the forested area burned in the seasonal snow zone, increasing at an average rate of up to 9% per year (*p* < 0.0001) with a total of 1.6% of the forested area burned in 2017 alone (Fig. 1). Not all 1980s and 1990s fires were mapped in Monitoring Trends in Burn Severity (MTBS)^16^, potentially resulting in a small overestimation in the trend. To assess the large-scale impacts of post-fire radiative forcing on snow, we used 2000–2016 moderate resolution imaging spectroradiometer (MODIS) satellite measurements of snow covered area across the American West to determine relative changes in snow disappearance date (SDD) before and after fire. This analysis (Methods) showed that for all studied fires in the seasonal snow zone throughout the West (*n* = 841), snow disappeared on average 5 days earlier as a result of post-fire radiative forcing (Fig. 2). The change in relative SDD was both immediate and persistent, with the relative disappearance date remaining constant for the first seven to eight years following fire but still significantly different from pre-fire conditions even after 10 years when our analysis ended.

The change in SDD (Fig. 2) suggests that forest fires impact the snow-dominated hydrology consistently throughout the American West for at least 10 years following fire. To assess the underlying causes of this persistence, we characterized the composition, magnitude, and duration of LAI in snow samples collected in early spring 2017 from a chronosequence of seven high-severity, pine-dominated burned forests (burned from 1 to 15 years prior to sampling) located in Wyoming, Utah, and Colorado, within the headwaters for the major rivers of the American West (i.e., Columbia, Missouri, Colorado, and Rio Grande; Fig. 1; Supplementary Table 1). We first used geochemical techniques^32,33^ to measure black carbon (BC; 0.09–0.6 µm) and dust (0.8–10 µm) concentrations, as well as conventional gravimetric techniques^34^ to measure organic and inorganic debris (>0.7 µm) concentrations in the snow samples. We then simulated the impact of measured LAI concentrations on snow albedo using the Snow, Ice, and Aerosol Radiation (SNICAR) model^35^. We also measured the spectral albedo directly on the snow samples. Post-fire radiative forcing on snow was calculated as the modeled difference in sunlight energy absorbed by an LAI-impacted (both SNICAR-modeled and directly measured albedo) and clean snowpack (with background levels of LAI concentrations), with the former assuming full snow surface irradiance and the latter with an assumed 60% reduction from full snow surface irradiance (Methods).

### Forest-fire-related impurities on snow

**Fig. 3:**
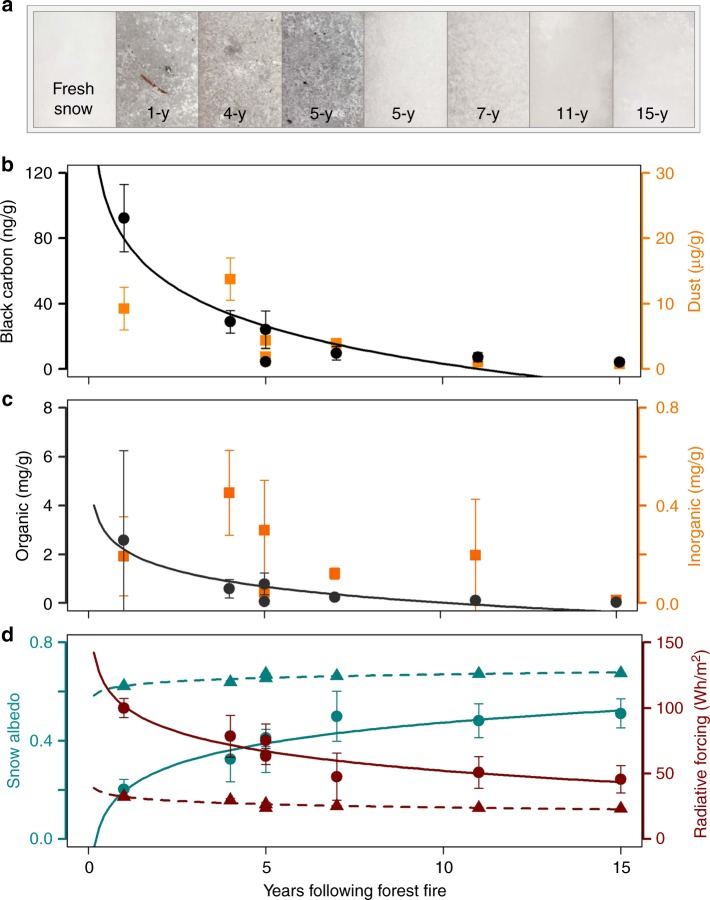
Changing impacts of light absorbing impurities (LAI) on snow following fire based on 2017 sampling of chronosequence of western burned forests (Fig. 1). **a** Photographs of snow-surface samples. **b** Black carbon and dust concentrations. **c** Organic and inorganic debris concentrations. **d** Measured (dark cyan dots and solid line) and SNICAR-modeled (dark cyan triangles and dashed line) snow albedo, with associated 15 January hourly, average, post-fire radiative forcing on snow for measured (dark red dots and solid line) and SNICAR-modeled (dark red triangles and dashed line) albedo values. Error bars indicate one standard deviation

During the first winter following fire, BC and organic debris were highly concentrated and both declined during the 15-year chronosequence (Fig. 3; BC, *R*^2^ = 0.84, *p* value < 0.01; organic debris, *R*^2^ = 0.83, *p* < 0.01). Conversely, dust including insoluble particles and inorganic debris showed no significant changes. BC and organic debris concentrations were most variable between sites on snowpacks from more recently burned forests, and BC concentrations measured in the burned forest 15 years after fire were similar to remote pre-industrial background levels of 0.5–5 ng g^−1^ measured in ice dated from 1750 to 1850 from the Upper Fremont Glacier, Wyoming^36^. Whereas dust concentrations were variable both within and between all burned forest sites, the greatest concentrations were from the southern Rocky Mountains, a region known for dust-on-snow events^37^. Inorganic debris concentrations measured in the chronosequence samples from the southern Rockies were similar to low-dust-year concentrations measured in subalpine snowpacks in the San Juan region of the Rocky Mountains^38^. Total gravimetric LAI measured at the San Juan region sites was attributed solely to atmospheric dust deposition on snow.

### Forest-fire-related solar forcing on snow

Both SNICAR-modeled and measured albedo were lowest during the first year following fire and increased during the 15-year chronosequence (Fig. 3; modeled; *R*^2^ = 0.81, *p* value < 0.01; measured, *R*^2^ = 0.88, *p* value < 0.01), from 0.62 to 0.68 and 0.2 to 0.51 for modeled and measured, respectively. We attribute the majority of SNICAR-modeled albedo change to BC concentrations (81% one winter following fire; 59% 15 winters following fire). Measured snow albedo incorporating impacts of both fine-grained (BC and dust) and course-grained (organic and inorganic) impurities showed greater changes than SNICAR-modeled snow albedo that included only fine-grained (BC and dust) impurities (Fig. 3). The difference between measured and simulated snow albedo values suggests that, in addition to BC and dust, larger impurities (e.g., micro-charcoal and burned woody debris) also make critical contributions to the snowpack energy balance and ultimately snowmelt.

**Fig. 4:**
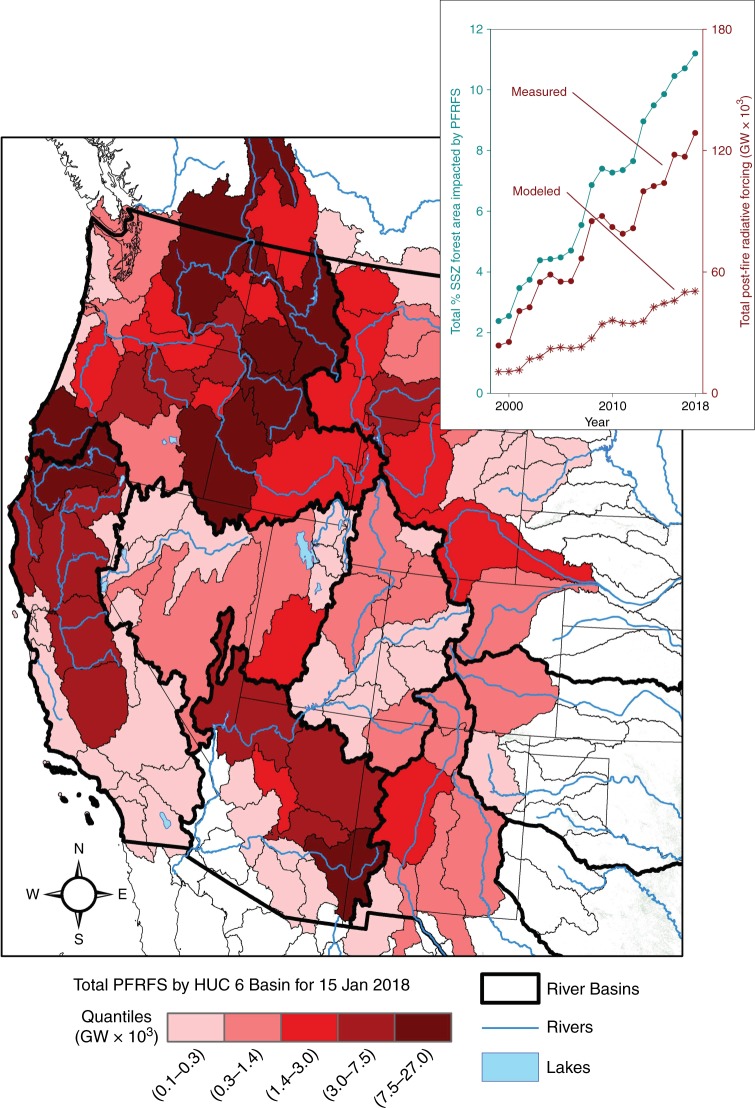
Total daily post-fire radiative forcing on snow (PFRFS) in the western U.S. for 15 January 2018 based on measured albedo values from 2017 chronosequence of burned forests, and change from 1999 through 2018 based on SNICAR-modeled (red dots) and measured (red stars) albedo values (inset). A 372% (modeled) to 443% (measured) increase in post-fire radiative forcing on snow occurred since 1999, and currently over 11% of all forests in the seasonal snow zone are experiencing enhanced radiative forcing due to forest fire occurrence within the last 15 years

We determined the radiative forcing on snow associated with the SNICAR-modeled and measured albedo values using solar insolation at 40°N latitude on January 15—the day of year corresponding to maximum North American snow-covered area^39^. Calculated radiative forcing was 32 and 101 Wh m^−2^ during the first winter following forest fire for the SNICAR-modeled and measured albedo values, respectively, declining to 23 and 44 Wh m^−2^ after 15 years (Fig. 3). Combining satellite-measured recent increases in annual burned area in forests (Fig. 1) with 15-year persistence of post-fire radiative forcing (Fig. 3) indicates a 366% recent increase in the extent of western snowpack impacted by post-fire radiative forcing (Fig. 4)—from 2.4% in 1999 to 11.2% in 2018. Using the more conservative SNICAR-modeled snow albedo values, total daily post-fire radiative forcing in the West increased 372% from 10.6 × 10^3^ GW in 1999 to 50.0 × 10^3^ GW in 2018. Using measured snow albedo values, total daily post-fire radiative forcing increased 443% from 23.7 × 10^3^ GW in 1999 to 128.7 × 10^3^ GW in 2018. For perspective, the 2018 forcing was 3.6 times the annual energy output of the Grand Coulee Dam (the largest energy producing dam in the U.S.), and sufficient energy to melt 1.4 billion m^3^ (1.1 million acre-feet) of ripe (i.e., isothermal at 0 °C) snowpack. Since 1984, however, 56% of forest area burned in the western seasonal snow zone occurred in the Columbia River Basin, the largest river by volume flowing into the Pacific Ocean from the western hemisphere, and the fourth largest river by volume in the U.S.^40^. Total daily 2018 post-fire radiative forcing on January 15 across the Columbia River Basin alone was sufficient to melt 0.82 billion m^3^ (0.66 million acre-feet) of ripe snowpack, equivalent to 213% of the daily discharge on 15 January or 0.35% of annual Columbia River discharge (average discharge at Vancouver, WA from 2007 to 2017). Although substantial, these estimates of total post-fire radiative forcing on snow are conservative because they were calculated on January 15 to correspond with maximum snow-cover extent but when insolation is low. The majority of seasonal snow melts later in spring when insolation and thus the magnitude of post-fire radiative forcing are larger.

## Discussion

Our results suggest that forest fires in the seasonal snow zone have immediate and profound impacts on snowmelt throughout burned forests in the American West, resulting in accelerated snow disappearance, earlier springtime meltwater release, and likely lower stream flows during drier summer months. Under climate warming, snowpack and associated water resource vulnerability will increase as forest fires become more frequent and extensive across the landscape^19,21,41,42^. As more snow falls as rain and less water is stored in warmer snowpacks, local forest fire effects related to earlier snowmelt also are likely to increase because, shallower snowpacks require less energy to melt, snow grain size is larger in warmer snowpacks which absorbs more sunlight energy, and shallower snowpacks accumulate greater impurity concentrations assuming no change in dry deposition of black carbon and burned organic debris. Human effects also contribute to snow-water resource vulnerability on a global scale, directly through aerosol emission/deposition of black carbon and dust on snowpack, and indirectly though greenhouse gas emissions, while simultaneously water resource demands are increasing for forest, agriculture, and urban use^41^. Forest fire effects to snow water resources are a concern for land managers and policy makers who have the common objective of optimizing consumption of natural resources while preserving the integrity of the landscape. Operational water models must include forest fire disturbance effects on snowpack if they are to accurately predict flood risks, drought potential, and downstream water resource availability.

## Methods

### Remote sensing analyses

To determine the area burned each year across the West, we used the MTBS fire perimeter data product that spans the 1984–2015 Landsat record at 30 m spatial resolution^43^. Note that not all the 1980s and 1990s fires were mapped in MTBS^16^, potentially introducing bias in the early part of the trend curve. In addition, we do not consider changes in fire activity prior to 1984 when Landsat 5 measurements began. For fires which occurred in 2016 and 2017 we used the Geospatial Multi-Agency Coordination Group fire perimeter data available online at http://rmgsc.cr.usgs.gov/outgoing/GeoMAC/historic_fire_data/. Here, we defined the western U.S. as the 11 western states of Arizona, California, Colorado, Idaho, Montana, Nevada, New Mexico, Oregon, Utah, Washington, and Wyoming. Within the perimeters of burned areas, we evaluated changes in burned forested area in the seasonal snow zone. Forested area was defined by tree density >20% in the LANDFIRE Existing Vegetation Cover (EVC) product^44^. The seasonal snow zone was determined using the MOD10A1 8-day snow-covered area product from MODIS as regions where snow was present in >25% of images centered on January 15 (2000–2017)^24^.

We evaluated the difference in SDD before and after forest fire for all forests burned between 2004 to 2010 in the western seasonal snow zone included in the MTBS database. The SDD product was developed from 2000 to 2016 Moderate Resolution Imaging Spectroradiometer (MODIS) MOD10A1 daily 500 m snow-cover data^45^ as the last day snow cover was observed (centered on the first five-day period without snow). To account for year-to-year changes in SDD resulting from meteorologic and geographic variability, we compared the SDD in forested areas inside each burn perimeter (n = 841) with that in a surrounding 2 km buffer. This difference in burned and unburned SDD (dSDD) was computed from 2000 to 2016 for all areas that burned between 2004 and 2010 to obtain at least five pre- and post-fire years. The pre-fire average dSDD was subtracted from each annual value to determine the change in dSDD before and after fire (Fig. 2). To account for inter-annual variability in forest cover, climate, and snowfall as well as satellite retrievals, we compared snowmelt in the burned forest to the surrounding unburned forest for 10 years of pre-fire conditions and found essentially no difference. This suggests that this approach to determining changes in SDD was valid, although we acknowledge potential biases in estimating snow cover from satellite imagery^45,46^. All spatial analyses were conducted in ArcInfo 10.4.1^47^, and all statistical analyses were conducted in R version 3.3.3^48^. Statistical relationships were tested for a significance level of 0.01.

### Field sampling

We sampled snow from a chronosequence of seven winter-accessible pine-dominated forests in the western seasonal snow zone that burned severely over 20 km^2^ within the past 15 years. These sites were located in Wyoming, Utah, and Colorado—within the headwaters of the largest volume rivers in the West including the Columbia, Colorado, and Rio Grande Rivers (Supplementary Table 1). At each research site, we identified three replicate snow sampling locations in high-severity burned forested areas of >1 km^2^ using Landsat-derived delta normalized burn ratio threshold value of >390^49^. At each sampling location, we collected a snow-surface sample, a snow-core sample of the entire snowpack, and measured snowpack properties, including snow water equivalent (SWE) and snow depth^24^. We collected the top 3 cm from a 0.5 m × 1 m plot on the snowpack surface using sterile scoops and Whirl-Pak® bags. Snow samples were kept frozen until analyzed in the laboratory. At each snow sampling location, we measured SWE using a federal snow sampler, and snow depth using a depth probe at nine randomly selected locations within a 50 m radius of each snow sample location to obtain a local average.

### Spectral albedo measurements

Prior to chemical and other analyses, we decontaminated the snow samples by removing the outer 1 cm of snow on all sides as well as scraped the tops and bottoms using a pre-cleaned ceramic knife. In our cold laboratory (−15 °C), we measured spectral albedo for each 1 nm band throughout the range from 350 to 2500 nm using an Analytical Spectral Devices® Full-Range Field Spectrometer (ASD-FR) with a contact probe mounted in an opaque Teflon^™^ light exclusion chamber that held the probe 1 mm above the snow sample. We made the spectral albedo measurements relative to a Spectralon® white reference target for every 10 cm section of each snow sample. A spectral correction was applied to the original measurements to correct for the observed offset as a result of an imperfect white reference. We then integrated the spectral albedo measurements across the spectral range and normalized by the illumination spectrum for each snow-surface sample.

### Geochemical analyses

After sample decontamination and spectral measurements, we divided the snow samples into three subsample sets and stored them in Whirl-Pak bags for subsequent geochemical analysis. To evaluate the mass of small light-absorbing impurities (LAI), including BC and crustal dust, one subsample set was analyzed using a continuous flow analysis system normally used for ice core measurements^32,33^. Samples were melted in Whirl-Pak bags, sonicated for 3 min, and transferred into pre-cleaned sample vials. Using an autosampler, samples were pumped through a 20 µm stainless steel filter to remove large particles (to prevent clogging the lines) and then into a low-volume debubbler^50^. From the debubbler, samples were split for measurements of BC in the size range 0.09–0.6 µm and insoluble particles used as a proxy for crustal dust in the size range 0.8–10 µm. We measured BC mass concentrations using a Single Particle Soot Photometer® (SP2; Droplet Measurement Technologies). SP2 measurements are based on incandescence. Instrument calibrations were conducted twice daily using three different standard concentrations. From replicate analyses of ice cores and snow samples, typical errors in concentration measurements are <5%^50–52^. Measurements of insoluble particle mass and size distribution were made using an Abakus® laser-based particle counter that determines semiquantitative, size-resolved particle counts^53^. Concentrations from the Abakus measurements were binned into four sizes based on requirements for the radiative transfer model (0.8–1, 1–2.5, 2.5–5, and 5–10 µm).

To verify that insoluble particle concentrations from the Abakus measurements were composed largely of crustal dust, we analyzed one subsample set for total concentrations of a broad range of elements^54^ using inductively coupled plasma mass spectrometry (ICP-MS; Thermo® Element 2). Samples were melted in Whirl-Pak bags and transferred into acid-cleaned sample vials in a class-100 clean room. Samples were then acidified to 1% HNO_3_ using concentrated ultrapure nitric acid and stored for 3 months^54^ prior to analysis. Ultrapure nitric acid spiked with indium was used as an external standard and introduced to the sample line just prior to sample injection into the ICP-MS instrument. Rare earth element (Ce, Dy, Gd, Pr) measurements made using the high-resolution ICP-MS confirmed that insoluble particles measured by the Abakus were composed largely of crustal dust (*r* = 0.68, *p* < 0.01).

To determine the mass and organic composition by weight of larger impurities (e.g., micro-charcoal or burned woody debris) on the snowpack surface, one subsample set was melted in Whirl-Pak bags and vacuum-filtered using Whatman® GF/F glass fiber filters (average pore space 0.7 µm). Using loss-on-ignition to distinguish organic vs. inorganic debris concentrations on snowpack^34^, the filters were combusted in a muffle furnace for 2 h at 530 °C and the organic debris calculated as the difference in mass before and after combustion.

### Radiative transfer modeling

To estimate the additional solar energy absorbed by a snowpack as a result of forest fire occurrence and subsequent deposition of LAI, we used BC and dust measurements in conjunction with the SNICAR model^35,55^ (available online at http://snow.engin.umich.edu/). SNICAR uses a two-stream radiative transfer solution^56^ to calculate snow albedo with known concentrations of BC and dust, snow-grain size, and incident-solar-flux characteristics. To isolate the impacts of LAI variability on radiative forcing from geographic location (e.g., slope, aspect, and latitude), we calculated the snow albedo for LAI-impacted and pristine snow using consistent parameters in SNICAR (i.e., direct radiation, effective grain size of 1500 µm, snowpack density of 300 kg m^−2^, average solar conditions for 15 January at 40°N latitude, including 4.225 MW m^−2^ per day, solar zenith angle of 61°). The largest snow-grain size in SNICAR was selected to represent the isothermal at 0 °C snowpack consistent with field observations. We used optical parameters for hydrophobic BC in SNICAR based on the assumption that the BC was relatively recently emitted and deposited within or a short distance from the burned forest. Because the radiative properties of larger LAI particles (e.g., micro-charcoal, burned woody debris) are not well understood and therefore not included in the SNICAR model, we also used direct spectral albedo measurements to estimate radiative forcing on snow.

Post-fire radiative forcing was calculated as the modeled difference in net snowpack shortwave radiation between the dirty (LAI-impacted) snow albedo values (both SNICAR-modeled and directly measured) and an equivalent clean snow albedo (with background levels of LAI concentrations). Background BC concentrations of 1 ng g^−1^ used in clean snow scenarios were derived from BC measurements in snow from the oldest (15-year old) fire, and were similar to pre-industrial background levels of BC in snow measured in ice dated from 1750 to 1850 from the Upper Fremont Glacier, Wyoming^36^. No background dust concentration was used in the clean snow scenarios because of the broad spatial variability in dust and that dust concentrations at each site were assumed to be unaffected by fire. A burned forest canopy with full snow surface irradiance was used for the LAI-impacted simulations. For the pristine snow simulations, we used an unburned forest canopy with a 60% reduction from full snow surface irradiance^24^. We assumed that the increase in incoming solar radiation as a result of the more open canopy was constant throughout the 15-year period of our analysis.

### Geospatial analyses

To evaluate temporal variability in the maximum impact of forest fire effects to snowpack across the West for the period 1984–2017, we determined the radiative forcing each year for January 15th which is the average date of maximum North American seasonal snow cover extent^39^. Post-fire radiative forcing on snow coefficients were developed using a logistical regression of the SNICAR-modeled and measured albedo, and associated post-fire radiative forcing values during the 15 years following fire. The annual total daily post-fire radiative forcing on snow each year was calculated using the SNICAR-modeled and measured radiative forcing coefficients for the 15 years following fire (Fig. 3), applied cumulatively for the total forested area burned in seasonal snow (Fig. 1) within 15 years following fire. The spatial distribution of the post-fire radiative forcing on snow was evaluated for 2018 by integrating the radiative forcing on snow in forests burned within 15 years for each HUC 6 basin in the American West (Fig. 4).

## Supplementary information

Supplementary Information

Source Data

## Data Availability

The authors declare that all data supporting the findings of this study are available within already existing public repositories, and the Source Data file. The MTBS data product can be found online at https://www.mtbs.gov/. The source data underlying Figs. 1a, b, 2, 3a–c, and 4a, b are provided as a Source Data file.

## Code availability

The snow disappearance date and post-fire radiative forcing on snow processing scripts are available from the authors upon request. The SNICAR radiative transfer modeling scripts are available from https://github.com/EarthSciCode/SNICARv2.

## References

[1] Serreze M, Clark M, Armstrong R, McGinnis D, Pulwarty R (1999). Characteristics of the western United States snowpack from snowpack telemetry (SNOTEL) data. Water Resour. Res..

[2] Buytaert W, Cuesta-Camacho F, Tobon C (2011). Potential impacts of climate change on the environmental services of humid tropical alpine regions. Glob. Ecol. Biogeogr..

[3] Tague, C. & Grant, G. Groundwater dynamics mediate low-flow response to global warming in snow-dominated alpine regions. *Water Resour. Res.* **45**, 10.1029/2008WR007179 (2009).

[4] Hunsaker CT, Whitaker TW, Bales RC (2012). Snowmelt runoff and water yield along elevation and temperature gradients in California’s southern Sierra Nevada. J. Am. Water Resour. Assoc..

[5] Pederson G, Betancourt J, McCabe G (2013). Regional patterns and proximal causes of the recent snowpack decline in the Rocky Mountains, US. Geophys. Res. Lett..

[6] Abatzoglou J (2011). Influence of the PNA on declining mountain snowpack in the Western United States. Int. J. Climatol..

[7] Stewart I, Cayan D, Dettinger M (2005). Changes toward earlier streamflow timing across western North America. J. Clim..

[8] Barnett TP, Adam JC, Lettenmaier DP (2005). Potential impacts of a warming climate on water availability in snow-dominated regions. Nature.

[9] Barnhart TB (2016). Snowmelt rate dictates streamflow. Geophys. Res. Lett..

[10] Harpold AA (2016). Diverging sensitivity of soil water stress to changing snowmelt timing in the western US. Adv. Water Resour..

[11] Winchell TS, Barnard DM, Monson RK, Burns SP, Molotch NP (2016). Earlier snowmelt reduces atmospheric carbon uptake in midlatitude subalpine forests. Geophys. Res. Lett..

[12] Steltzer H, Landry C, Painter TH, Anderson J, Ayres E (2009). Biological consequences of earlier snowmelt from desert dust deposition in alpine landscapes. Proc. Natl Acad. Sci..

[13] Cox CJ (2017). Drivers and environmental responses to the changing annual snow cycle of northern Alaska. Bull. Am. Meteorol. Soc..

[14] Balch JK (2017). Human-started wildfires expand the fire niche across the United States. Proc. Natl Acad. Sci..

[15] Westerling A, Hidalgo H, Cayan D, Swetnam T (2006). Warming and earlier spring increase western US forest wildfire activity. Science.

[16] Dennison P, Brewer S, Arnold J, Moritz M (2014). Large wildfire trends in the western United States, 1984-2011. Geophys. Res. Lett..

[17] Williams CJ, Pierson FB, Robichaud PR, Boll J (2014). Hydrologic and erosion responses to wildfire along the rangeland–xeric forest continuum in the western US: a review and model of hydrologic vulnerability. Int. J. Wildland Fire.

[18] Moritz, M. et al. Climate change and disruptions to global fire activity. *Ecosphere* **3**, 10.1890/ES11-00345.1 (2012).

[19] Westerling A (2011). Climate change and growth scenarios for California wildfire. Clim. Change.

[20] Littell J, McKenzie D, Peterson D, Westerling A (2009). Climate and wildfire area burned in western U. S. ecoprovinces, 1916-2003. Ecol. Appl..

[21] Council, N. R. *Climate Stabilization Targets: Emissions, Concentrations, and Impacts Over Decades to Millennia*. (National Academies Press, Washington DC, 2011).

[22] Burles K, Boon S (2011). Snowmelt energy balance in a burned forest plot, Crowsnest Pass, Alberta, Canada. Hydrol. Process..

[23] Winkler RD (2011). Changes in snow accumulation and ablation after a fire in south-central British Columbia. Streamline Watershed Manag. Bull..

[24] Gleason K, Nolin A, Roth T (2013). Charred forests increase snowmelt: effects of burned woody debris and incoming solar radiation on snow ablation. Geophys. Res. Lett..

[25] Gleason K, Nolin A (2016). Charred forests accelerate snow albedo decay: parameterizing the post-fire radiative forcing on snow for three years following fire. Hydrol. Process..

[26] Stevens JT (2017). Scale-dependent effects of post-fire canopy cover on snowpack depth in montane coniferous forests. Ecol. Appl..

[27] Harpold AA (2014). Changes in snow accumulation and ablation following the Las Conchas Forest Fire, New Mexico, USA. Ecohydrology.

[28] Micheletty P, Kinoshita A, Hogue T (2014). Application of MODIS snow cover products: wildfire impacts on snow and melt in the Sierra Nevada. Hydrol. Earth Syst. Sci..

[29] Maxwell JD, Call A, Clair SBS (2019). Wildfire and topography impacts on snow accumulation and retention in montane forests. For. Ecol. Manag..

[30] Hallema DW (2018). Burned forests impact water supplies. Nat. Commun..

[31] Kinoshita AM, Hogue TS (2015). Increased dry season water yield in burned watersheds in Southern California. Environ. Res. Lett..

[32] McConnell J, Aristarain A, Banta J, Edwards P, Simoes J (2007). 20th-Century doubling in dust archived in an Antarctic Peninsula ice core parallels climate change and desertification in South America. Proc. Natl Acad. Sci. USA.

[33] McConnell J (2007). 20th-century industrial black carbon emissions altered arctic climate forcing. Science.

[34] Dean Jr, W. E. Determination of carbonate and organic matter in calcareous sediments and sedimentary rocks by loss on ignition: comparison with other methods. *J. Sediment. Res.* **44**, 242–248 (1974).

[35] Flanner, M., Zender, C., Randerson, J. & Rasch, P. Present-day climate forcing and response from black carbon in snow. *J. Geophys. Res.* **112**, 10.1029/2006JD008003 (2007).

[36] Chellman N (2017). Reassessment of the upper fremont glacier ice-core chronologies by synchronizing of ice-core-water isotopes to a nearby tree-ring chronology. Environ. Sci. Technol..

[37] Painter T (2010). Response of Colorado River runoff to dust radiative forcing in snow. Proc. Natl Acad. Sci. USA.

[38] Skiles S, Painter T (2017). Daily evolution in dust and black carbon content, snow grain size, and snow albedo during snowmelt, Rocky Mountains, Colorado. J. Glaciol..

[39] Robinson DA, Frei A (2000). Seasonal variability of Northern Hemisphere snow extent using visible satellite data. Prof. Geogr..

[40] Kammerer, J. C. Largest rivers in the United States (water fact sheet). Report No. 2331–1258 (US Geological Survey, Washington, DC, 1987).

[41] Barnett TP (2008). Human-induced changes in the hydrology of the western United States. Science.

[42] Nolin A, Daly C (2006). Mapping “at risk” snow in the Pacific Northwest. J. Hydrometeorol..

[43] Eidenshink J (2007). A project for monitoring trends in burn severity. Fire Ecol..

[44] Rollins MG (2009). LANDFIRE: a nationally consistent vegetation, wildland fire, and fuel assessment. Int. J. Wildland Fire.

[45] Hall DK, Riggs GA, Salomonson VV, DiGirolamo NE, Bayr KJ (2002). MODIS snow-cover products. Remote Sens. Environ..

[46] Parajka, J. & Blöschl, G. Spatio‐temporal combination of MODIS images—potential for snow cover mapping. *Water Resour. Res.* **44**, W03406 (2008).

[47] ESRI. (Environmental System Research Institute, Redlands, CA, 2015).

[48] Team, R. C. (R Foundation for Statistical Computing Vienna, Austria, 2016).

[49] Miller J, Thode A (2007). Quantifying burn severity in a heterogeneous landscape with a relative version of the delta Normalized Burn Ratio (dNBR). Remote Sens. Environ..

[50] Macdonald K (2017). Observations of atmospheric chemical deposition to high Arctic snow. Atmos. Chem. Phys..

[51] Bisiaux M (2012). Changes in black carbon deposition to Antarctica from two high-resolution ice core records, 1850–2000 AD. Atmos. Chem. Phys..

[52] Arienzo, M. et al. Holocene black carbon in Antarctica paralleled Southern Hemisphere climate. *J. Geophys. Res.* **122**, 6713–6728 (2017).

[53] Ruth, U., Wagenbach, D., Steffensen, J. P. & Bigler, M. Continuous record of microparticle concentration and size distribution in the central Greenland NGRIP ice core during the last glacial period. *J. Geophys. Res.* **108**, 4098 (2003).

[54] Uglietti C, Gabrielli P, Olesik JW, Lutton A, Thompson LG (2014). Large variability of trace element mass fractions determined by ICP-SFMS in ice core samples from worldwide high altitude glaciers. Appl. Geochem..

[55] Flanner M (2009). Springtime warming and reduced snow cover from carbonaceous particles. Atmos. Chem. Phys..

[56] Toon OB, McKay C, Ackerman T, Santhanam K (1989). Rapid calculation of radiative heating rates and photodissociation rates in inhomogeneous multiple scattering atmospheres. J. Geophys. Res..

