# Supplementary material for: Four-fold increase in solar forcing on snow in western U.S. burned forests since 1999
Source: Nat Commun. 2019 May 2;10:2026. doi: 10.1038/s41467-019-09935-y (PMC6497640; doi:10.1038/s41467-019-09935-y)
Supplement: Supplementary file 1 — Supplementary Information [file 41467_2019_9935_MOESM1_ESM.pdf]

**Supplementary Information**

Four-fold increase in solar forcing on snow in western U.S. burned forests since 1999

Gleason et al.

Supplementary Table 1. Research sites represented a chronosequence of burned forests in seasonal snow, and were located in headwaters of the major western US rivers.

| **Fire ID** | **Year burned** | **Years-since-fire** | **State** | **River Basin** | **Lat.** | **Lon.** | **Sample day 2017** |
|---|---|---|---|---|---|---|---|
| Cliff Creek | 2016 | 1 | WY | Columbia | 43°14'25.37"N | 110°29'38.28"W | 22 Mar |
| West Fork | 2013 | 4 | CO | Colorado | 37°34'19.41"N | 106°47'56.18"W | 27 Mar |
| Church Camp | 2012 | 5 | UT | Colorado | 39°54'25.10"N | 110°41'56.94"W | 29 Mar |
| Horsethief Canyon | 2012 | 5 | WY | Columbia | 43°25'17.17"N | 110°45'21.56"W | 23 Mar |
| Bull | 2010 | 7 | WY | Columbia | 43°18'18.98"N | 110°31'17.03"W | 24 Mar |
| Mato Vega | 2006 | 11 | NM | Rio Grande | 35°36'27.2"N | 105°15'13.73"W | 26 Mar |
| Price Canyon | 2002 | 15 | UT | Colorado | 39°46'38.37"N | 110°54'6.66"W | 28 Mar |
